# Real-World Experience with Brolucizumab in Treatment-Naïve nAMD with Low Baseline Visual Acuity: Short-Term Outcomes from a Prospective Single-Institution Study

**DOI:** 10.3390/life16050754

**Published:** 2026-05-01

**Authors:** Arsim Hajdari, Valdet Uka

**Affiliations:** 1 Italian Eye Hospital, 10000 Prishtina, Kosovo; dr.arsimhajdari@gmail.com; 2 Department of Life Sciences, University of Business and Technology, 10000 Prishtina, Kosovo; 3 Division of Pharmacy, Faculty of Medicine, University of Prishtina, 10000 Prishtina, Kosovo

**Keywords:** nAMD, anti-VEGF, brolucizumab, real-world data, treatment-naïve, low visual acuity

## Abstract

Background: Neovascular age-related macular degeneration (nAMD) is a progressive chronic disease that represents a major cause of irreversible vision loss worldwide. In this study we aim to assess the short-term functional and anatomical outcomes of brolucizumab therapy in treatment-naïve patients with nAMD presenting with low baseline visual acuity in a single institution setting. Methods: This is a prospective non-randomized study that included 154 treatment-naïve eyes with low baseline visual acuity. We measured visual outcomes (BCVA, logMAR) and structural outcomes (CST, μm). We also stratified the study population into respective age subgroups to evaluate any possible trend between outcome changes and age differences. BCVA and CST were measured at baseline, at each consecutive month (month 1, 2 and 3) of the loading phase, as well as at the final timepoint (6 months). Intraocular pressure (IOP) before and after injection, as well as the incidence of serious adverse events, were monitored throughout the study. Results: Mean BCVA improved by 0.41 logMAR (+20 ETDRS letters) after the first injection, 0.65 logMAR (+32 letters) after the second, and reached a maximum improvement of 0.80 logMAR after the third injection. The most important BCVA improvement was seen in younger patients (<50 years), with mean BCVA decreasing from approximately 1.0 logMAR at baseline to around 0.3–0.4 logMAR at the final measurement. Mean CST declined by 45.5 μm after the first injection, 78.5 μm after the second, 117.8 μm after the third, and 143.6 μm at the final timepoint, indicating a pronounced anatomical response to intravitreal brolucizumab therapy. Conclusions: In conclusion, this study demonstrates that brolucizumab therapy provides significant short-term anatomical and functional improvements in treatment-naïve patients with nAMD and poor baseline visual acuity. Baseline visual acuity, treatment-naïve status, and patient age appear to be key determinants of visual gain.

## 1. Introduction

Neovascular eye diseases comprise a significant cause of irreversible blindness worldwide. This category of ophthalmic diseases is mainly characterized by pathological angiogenesis primarily controlled by the molecular signaling cascade of vascular endothelial growth factor (VEGF) [1,2]. These conditions include neovascular age-related macular degeneration (nAMD), diabetic macular edema (DME), retinal vein occlusion (RVO), and myopic choroidal neovascularization (mCNV), among which nAMD (also known as “wet” AMD) as a chronic progressive disease remains the principal cause of severe visual impairment in elderly populations in developed countries [3,4]. Current estimates show that nAMD affects around 200 million people worldwide and its prevalence is increasing constantly as the population ages [5]. The main pathological features of nAMD include the development of choroidal neovascularization, growth of abnormal blood vessels resulting in retinal exudation and subsequent fluid accumulation, hemorrhage, edema and progressive fibrous scarring, ultimately leading to central vision loss [6]. The discovery and development of intravitreal anti-VEGF therapy has certainly revolutionized the treatment of neovascular retinal diseases, resulting in significant improvements in visual acuity, subsequently reducing the risk of permanent vision loss [7,8,9]. However, the requirement for frequent intravitreal injections (IVIs), incomplete therapeutic response in a subset of patients, and the long-term treatment burden remain major clinical challenges for the patients as well as for the healthcare system [10,11]. Consequently, newer anti-VEGF drugs, such as brolucizumab and faricimab, have been formulated to achieve sustained VEGF inhibition, and allow for extended treatment regimens [12,13,14,15,16].

Brolucizumab (Beovu^®^, Novartis AG, Basel, Switzerland) is a humanized single-chain antibody fragment (scFv; 26 kDa) that selectively binds and inhibits all isoforms of VEGF-A, thereby suppressing the angiogenesis and permeability events key to neovascular ophthalmic diseases [17]. Pharmaceutically, it is an innovative anti-VEGF agent, currently approved in the US and EU by respective regulatory agencies in treating nAMD [18,19] and DME [20,21]. No biosimilars or additional indications have been reported yet. Possessing a 26 kDa molecular weight, brolucizumab is considered significantly smaller than ranibizumab (48 kDa), aflibercept (115 kDa) and faricimab (150 kDa). Owing to its small molecular size and higher solubility, brolucizumab enables delivery of a high molar dose (6 mg/0.05 mL) per IVI, potentially allowing enhanced tissue penetration and more prolonged VEGF suppression compared with larger anti-VEGF molecules. In the pivotal HAWK and HARRIER phase III trials, brolucizumab demonstrated non-inferior best-corrected visual acuity (BCVA) gains compared with aflibercept at week 48, with superior fluid resolution and a greater proportion of eyes maintained on 12-week dosing intervals (q12w) following the loading phase [12,22]. Moreover, its solid anatomical efficacy in reducing the central subfield thickness (CST) has been proved and further supported by robust reductions in intraretinal and subretinal fluid. In this context, recent real-world comparative studies have further highlighted the rapid anatomical and functional response associated with brolucizumab therapy. For instance, a study comparing brolucizumab with aflibercept demonstrated pronounced early reductions in retinal fluid and improvements in structural and vascular retinal parameters, supporting the potential for enhanced early disease control with brolucizumab [23]. Despite the demonstrated efficacy and durability of brolucizumab in randomized clinical trials, evidence from real-world data specifically addressing its performance in treatment-naïve patients presenting with low baseline visual acuity remains limited [24,25,26].

Although anti-VEGF therapy has proven a well-established efficacy, the majority of randomized clinical trials and real-world studies have primarily included patients with moderate baseline visual acuity, leaving a significant gap in evidence for those presenting with severely impaired vision. In particular, data on early functional and anatomical response in treatment-naïve patients with low baseline visual acuity remain limited. This subgroup represents a clinically important population, as treatment response dynamics and visual prognosis may differ substantially from those with better baseline vision. Therefore, the present study aims to evaluate the short-term functional and anatomical outcomes of intravitreal brolucizumab in treatment-naïve patients with nAMD and low baseline visual acuity in a real-world clinical setting, with particular emphasis on early treatment response.

## 2. Materials and Methods

### 2.1. Study Design and Ethical Considerations

This is a prospective, single-center, non-randomized longitudinal study conducted at the Italian Eye Hospital in Prishtina, Kosovo, which includes data from patients with nAMD receiving intravitreal brolucizumab injections between May 2023 and December 2024. All procedures and research activities conducted during this study were in full accordance with the Declaration of Helsinki and complied with applicable domestic regulations in Kosovo. The Italian Eye Hospital granted the respective ethical permission for the foreseen research (Ref. ID No.: 811489550; dt. 28 April 2023). Informed consent was obtained from all subjects involved in the study. Participants could choose to leave the study at any time. All data were appropriately anonymized.

### 2.2. Participants and Eligibility Criteria

In total, 154 eyes from 150 newly diagnosed (treatment-naïve) nAMD patients were part of the study and were followed up for 6 months. Inclusion criteria were age >18 years, diagnosis of nAMD, and treatment-naïve patients with low visual acuity (≤0.4 decimal Snellen scale). A cutoff of ≤0.4 Snellen allowed us to focus on a clinically relevant cohort of patients with reduced visual function; however, we are aware that use of a predefined visual acuity cutoff may limit direct comparability with other studies applying different inclusion criteria. When both eyes fulfilled the inclusion criteria, both were included in the study. Exclusion criteria were denial of informed consent, macular scarring preventing a change in vision, patients previously treated with other anti-VEGF agents, patients with other ophthalmic diseases (e.g., co-existing vitreoretinal pathologies other than nAMD, glaucoma, uveitis, optic nerve diseases, etc.), patients with significant media opacities that prevent observation of ocular fundus, patients with previous history of retinal surgery, and patients with any history of systemic vasculitis and/or autoimmune diseases. Smoking status was assessed based on patient self-report and recorded as a binary variable (yes/no).

### 2.3. Ophthalmic Examination

At every visit, patients were subjected to detailed ophthalmic examination including intraocular pressure (IOP), slit-lamp examination (SLE), dilated ocular fundus examination, BCVA assessment and optical coherence tomography (OCT) imaging. IOP was assessed using non-contact tonometry with an air-puff tonometer (NCT-200, Reichert Technologies, Depew, NY, USA). SLE of the anterior segment was performed using a slit-lamp biomicroscope (Haag-Streit AG, Köniz, Switzerland). Retinal structure was quantitatively assessed with CST as a primary anatomical outcome, using spectral-domain optical coherence tomography (SD-OCT) (3D OCT-2000 FA Plus, Topcon Corporation, Tokyo, Japan). OCT angiography (OCTA) was performed as part of routine clinical evaluation to qualitatively assess retinal and choroidal vasculature (Topcon Corp., Tokyo, Japan). However, no quantitative OCTA-derived parameters were included in the analysis. All imaging was performed using standardized macular scan acquisition protocols according to the manufacturer’s recommendations throughout the study period. Patients were advised to report promptly any discomfort or suspicious adverse reaction throughout the study. Thus, detailed history regarding any ocular or systemic adverse effects was recorded at each visit.

### 2.4. Intravitreal Injection Procedure

Intravitreal injections (IVIs) were performed under aseptic conditions in the operating room. The ocular surface was first disinfected with 5% povidone iodine solution, followed by topical anesthesia with 0.5% tetracaine hydrochloride eye drops. Injections were carried out using the standard pars plana approach, 4 mm posterior to the limbus using a 30-gauge needle and pre-filled syringes of brolucizumab (6 mg/0.05 mL) (Beovu, Novartis). Post-injection care included topical TobraDex^®^ (tobramycin 0.3%/dexamethasone 0.1%; Alcon-Couvreur N.V., Puurs, Belgium) eye drops every 6 h and Dicloftil^®^ (Diclofenac sodium, 0.1%; Farmigea S.p.A., Pisa, Italy) eye drops every 8 h for two weeks. Patients with bilateral indication (4 patients) received the IVI on different days for each eye. All the IVIs were performed by the same ophthalmologist (A.H.).

### 2.5. Outcome Measures

**Figure 1:**
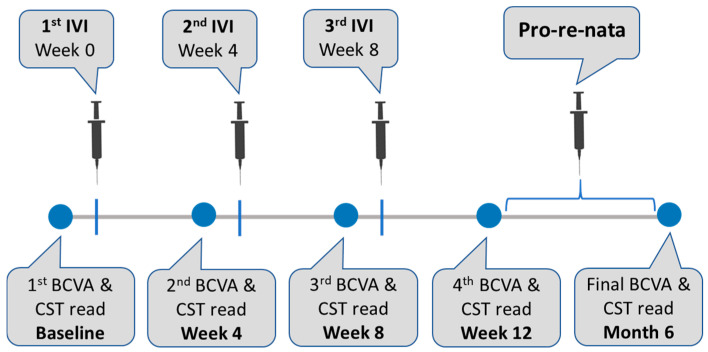
Intravitreal injection (IVI) protocol of brolucizumab in this study and the schedule of the outcome measures. Abbreviations: BCVA (best-corrected visual acuity), CST (central subfield thickness).

We measured visual outcomes (BCVA) and structural outcomes (CST). The main outcome measure was BCVA, which was measured using the Snellen decimal scale and then converted to logarithmic of the minimum angle resolution (LogMAR) values for statistical analysis. On the other hand, CST as an anatomical outcome was assessed using SD-OCT and defined as the mean retinal thickness (in μm) between the internal limiting membrane and Bruch’s basement membrane within the central fovea. Both outcome measures (BCVA and CST) were measured at five different timepoints: at baseline, after each of the three consecutive IVIs of the loading phase, as well as at the final timepoint (Figure 1). We also monitored IOP for each patient before the IVI and 24 h after the IVI.

### 2.6. Statistical Analysis

Statistical analyses were performed to evaluate longitudinal changes in BCVA (logMAR) and CST following intravitreal brolucizumab therapy. We also statistically evaluated the IOP before and after each IVI. Continuous variables were summarized as mean ± 95% confidence interval (CI), unless explicitly indicated in a different way. Changes over time were analyzed using repeated-measures analysis of variance (ANOVA), with timepoint treated as a within-subject factor since the same patients were measured multiple times. Normality of residuals was assessed using both visual inspection (histograms and *qq* plots) and the Shapiro–Wilk test. Given the relatively large sample size, visual methods were prioritized due to the sensitivity of formal tests to minor deviations from normality. The assumption of sphericity was evaluated using Mauchly’s test, and when violated, Greenhouse–Geisser (GG) correction was applied to adjust degrees of freedom. Effect sizes were calculated using generalized eta-squared (ges) to quantify the magnitude of treatment effects. Post hoc pairwise comparisons between timepoints were performed using estimated marginal means with Holm–Bonferroni correction for multiple testing. Aiming to find a relationship between reduction in CST and improvement in BCVA, Pearson correlation statistics were applied. For IOP differences before and after each IVI, a paired *t*-test was used. In all cases, a *p* value < 0.05 was considered statistically significant. All statistical analyses and data visualizations were carried out using *R* programing (Version 4.5.2; Foundation for Statistical Computing, Vienna, Austria) [27]. No formal a priori sample size or power calculation was performed, as this study was designed as a prospective real-world observational cohort. The sample size was determined by the number of consecutive eligible treatment-naïve patients presenting during the study period, reflecting routine clinical practice.

## 3. Results

A total of 154 eyes with neovascular AMD were included in the study. The mean age was 68.7 ± 8.3 years (mean ± SD), and 62.0% were male. The treated eye was the right eye in 48.7% while 2.6% had bilateral involvement. Current smokers represented 37.3% of the cohort. At baseline, the mean BCVA was 1.26 ± 0.36 logMAR (mean ± SD), and the mean CST was 476.6 ± 117.7 µm (mean ± SD). Demographic and baseline data are presented in Table 1. Repeated-measures ANOVA showed a significant improvement in BCVA (logMAR) across brolucizumab injections (F(2.44, 364.18) = 547.40, *p* < 0.001, ges = 0.563). Post hoc pairwise comparisons with Holm correction revealed an important change in BCVA at all follow-up visits compared to baseline (all *p* < 0.001). Mean visual acuity improved by 0.41 logMAR (+20 ETDRS letters) after the first IVI, 0.65 logMAR (+32 letters) after the second, and reached a maximum improvement of 0.80 logMAR after the third injection.

**Figure 2:**
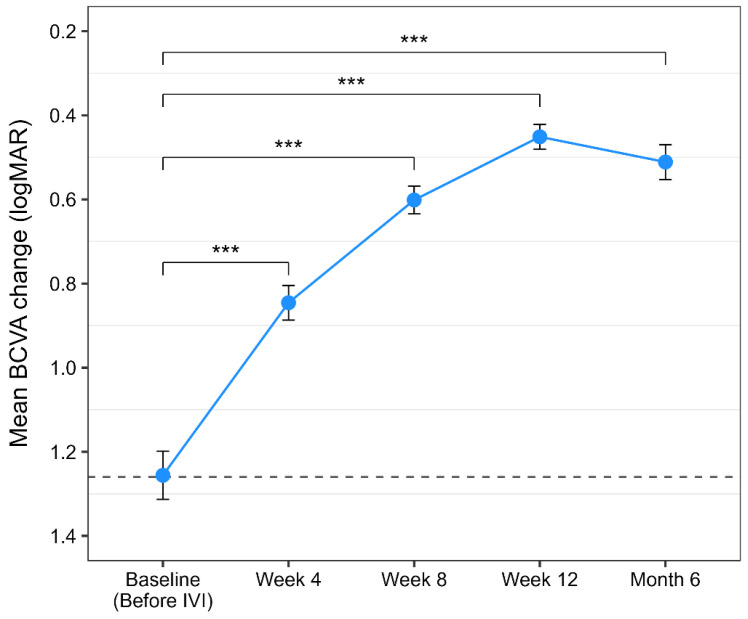
Mean BCVA (best-corrected visual acuity, logMAR) change over time with brolucizumab therapy. Points represent mean values, and error bars indicate the 95% confidence intervals. Stars indicate a statistically significant *p* value (*p* < 0.05). Dashed horizontal line indicates baseline mean value.

A small but statistically significant reduction in visual acuity was observed between the third IVI and the final BCVA measurement at 6 months follow-up (Δ = −0.06 logMAR, −3 letters; *p* = 0.004), indicating saturation of treatment response, while visual acuity remained significantly improved compared to baseline. The aforementioned BCVA results after brolucizumab therapy are depicted in Figure 2.

**Figure 3:**
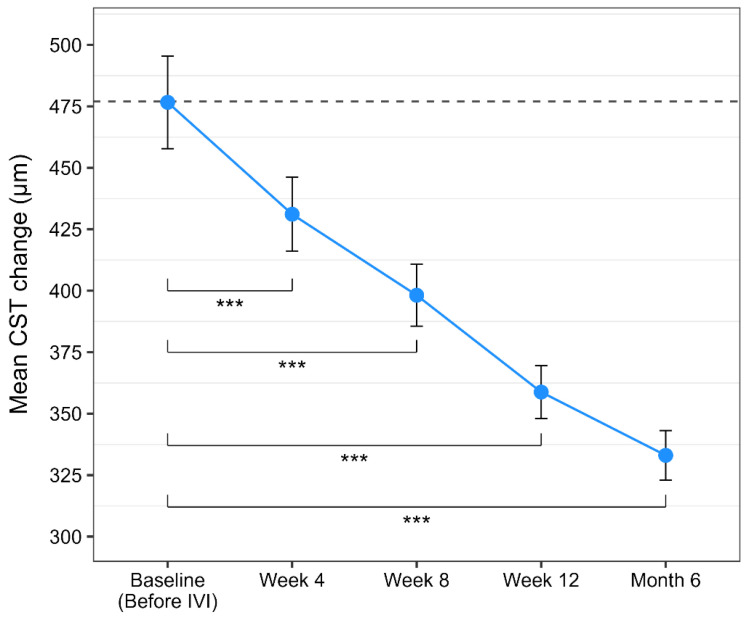
Mean CST (central subfield thickness, μm) change over time with brolucizumab therapy. Points represent mean values, and error bars indicate the 95% confidence intervals. Stars indicate a statistically significant *p* value (*p* < 0.05). Dashed horizontal line indicates baseline mean value.

Analogically to BCVA, repeated-measures ANOVA revealed a significant reduction in CST across treatment visits (F(1.34, 199.45) = 313.17, *p* < 0.001, ges = 0.260), with a large effect size. These findings demonstrate a solid anatomical response that clearly corroborates with the potent anti-VEGF activity of brolucizumab. Post hoc pairwise comparisons demonstrated a significant reduction in central subfield thickness (CST) at all timepoint measurements compared with baseline (all *p* < 0.0001). Mean CST decreased by 45.5 μm after the first injection, 78.5 μm after the second, 117.8 μm after the third, and 143.6 μm at the final timepoint, indicating a progressive and sustained anatomical response to intravitreal brolucizumab therapy. Significant reductions were also noticed between subsequent IVIs, reflecting continued resolution of macular edema with ongoing treatment. CST results are visualized in Figure 3. A cumulative representation of post hoc results for both outcome measures (BCVA and CST) is depicted in Table 2. Pearson correlation analysis demonstrated a weak but statistically significant positive association between CST reduction and improvement in visual acuity (r = 0.20, 95% CI 0.04–0.35, *p* = 0.017).

**Figure 4:**
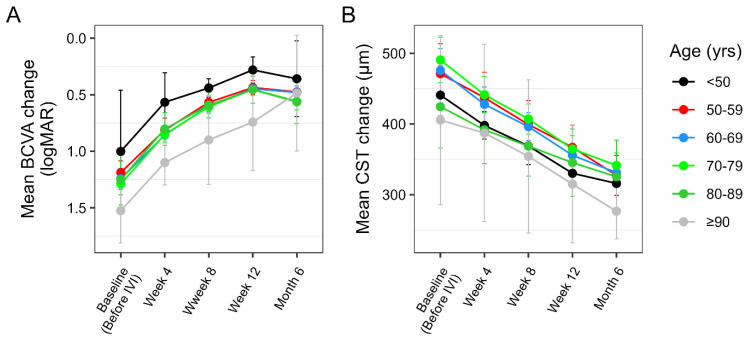
Mean BCVA (best-corrected visual acuity), logMAR (**A**), and mean CST (central subfield thickness, μm (**B**), change over time with brolucizumab therapy according to different age groups. Points represent mean values, and error bars indicate the 95% confidence intervals.

When stratified by age, all subgroups demonstrated improvement in mean BCVA following initiation of intravitreal brolucizumab therapy (Figure 4A). The most important functional improvement was seen in younger patients (<50 years), with mean BCVA decreasing from approximately 1.0 logMAR at baseline to around 0.3–0.4 logMAR at the final measurement.

Comparable improvements were observed in the 50–59, 60–69, and 70–79-year groups, which showed a steady decline in logMAR values over the treatment period. In contrast, the oldest patient subgroup (≥90 years) demonstrated a more modest response, most likely due to higher baseline logMAR values resulting in smaller reductions during follow-up, suggesting a less pronounced visual gain in this subgroup. Overall, the data indicate that while brolucizumab therapy was associated with improved visual acuity across all age categories, younger patients tended to achieve greater and more rapid functional improvement, whereas the oldest subgroup exhibited an attenuated improvement. These subgroup analyses are descriptive and exploratory in nature, and no formal statistical comparisons between age groups were performed.

In the same fashion as for BCVA, we also stratified the CST data by age (Figure 4B). In each subgroup, CST values declined consistently from baseline after successive IVIs, indicating a gradual anatomical improvement over the course of brolucizumab therapy. While baseline CST values varied moderately between age categories, the overall decreasing trend was comparable among groups. Slightly greater reductions in CST appeared to occur in younger and middle-aged subgroups, whereas the ≥90-year group showed somewhat smaller changes; however, there is no clear-cut distinction as to which group benefits the most and if there is logic in the trend of improvement.

**Figure 5:**
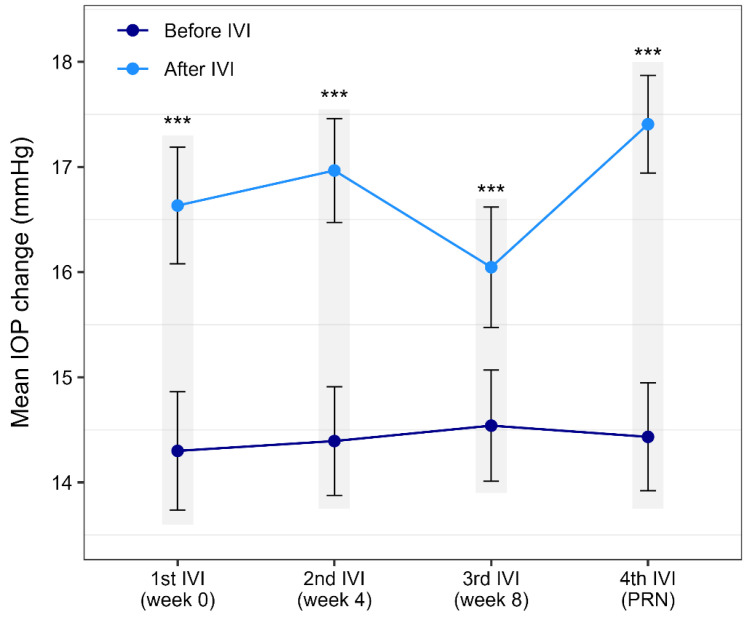
Intraocular pressure (IOP, mmHg) before and after brolucizumab IVIs. Points represent mean values, and error bars indicate the 95% confidence intervals. Stars indicate a statistically significant *p* value (*p* < 0.05) for each IVI (highlighted rectangle). IOP was measured before and 24 h after IVI. Abbreviations: IVI (intravitreal injection), PRN (pro-re-nata regimen).

On the other hand, statistical analysis (paired *t*-test) showed a significant increase in IOP after each IVI (all *p* < 0.001). The mean difference in IOP before and after the first IVI is 2.33 mmHg, whereas it reaches the maximal difference in the fourth IVI with 2.97 mmHg. The IOP fluctuations around each IVI are depicted in Figure 5.

No serious adverse events such as intraocular inflammation (IOI), endophthalmitis, vasculitis or other significant ocular and/or systemic adverse effects were observed throughout the brolucizumab therapy in the study cohort. However, general adverse effects related to anti-VEGF therapy such as conjunctival hemorrhage, eye irritation and iritis were encountered to some extent among the participants.

## 4. Discussion

In this prospective real-world study, we evaluated short-term functional and anatomical outcomes (6 months) of brolucizumab therapy in treatment-naïve patients with nAMD presenting with low baseline visual acuity. This is a subgroup that remains underrepresented in both clinical trials and real-world studies. Our findings demonstrate significant improvements in BCVA and CST, with the most pronounced functional gains occurring during the loading phase (first three IVIs; see Figure 1). In addition, the most rapid increment in visual gains is noticed after the first injection. The solid visual acuity gains observed in this cohort could be explained by the very poor baseline visual acuity (1.26 ± 0.36 logMAR), allowing for substantial functional improvement following treatment. In most anti-VEGF studies, eyes with lower baseline BCVA tend to show larger average visual acuity gains, most likely due to the ceiling effect of the therapy [28,29,30,31]. However, this does not hold true entirely regarding the final visual acuity which usually has a worse prognosis in patients with low baseline BCVA. Hence, despite the substantial improvement in BCVA observed in our cohort, the final visual acuity remained relatively limited, reflecting the impact of poor baseline vision. In this context, our results reinforce the importance of early diagnosis and timely initiation of anti-VEGF therapy. Importantly, these findings help address the limited evidence available for patients with poor baseline vision, providing real-world insight into early treatment response in this clinically challenging subgroup. The relatively poor baseline visual acuity observed in this cohort primarily reflects the predefined inclusion criteria. Nevertheless, delayed presentation may also contribute to reduced baseline vision in real-world settings, especially in developing countries like Kosovo where several factors such as lack of symptom awareness, low accessibility and affordability for specialized care, and inadequate referral patterns may have an important influence. In addition, our cohort is composed of treatment-naïve eyes which further supports the observed result regarding the significant increase in BCVA. There is plenty of evidence demonstrating that treatment-naïve eyes respond better to anti-VEGF therapy [32,33,34]. Moreover, the rapid functional and anatomical response observed in our cohort is consistent with previous reports describing a solid early treatment effect of brolucizumab compared to other agents such as faricimab [34]. However, direct comparisons with other agents such as faricimab should be interpreted cautiously due to limited head-to-head evidence. As was demonstrated previously in head-to-head pivotal HAWK and HARRIER phase-three trials, brolucizumab showed superiority to aflibercept in reducing CST and retinal fluid rates; in our data too, brolucizumab demonstrates a pronounced anatomical effect with an average reduction in retinal thickness of −143 μm at the final timepoint [12]. With regard to other anti-VEGF drugs such as ranibizumab or bevacizumab, brolucizumab’s advantage in anatomical improvements cannot be confirmed conclusively due to limited head-to-head comparative studies; however, indirect comparisons and network meta-analysis indicate this feature to a certain extent [35,36,37]. Better anatomical outcomes for brolucizumab are mainly attributed to its relatively low molecular weight, better solubility and higher molarity which ultimately translate to improved tissue penetration. In other words, inner retinal layers are better exposed to anti-VEGF inhibition with brolucizumab compared to other anti-VEGF agents [38].

The correlation between the reduction in CST and improvement in visual acuity indicates that greater anatomical drying was associated with greater functional gain. However, the modest strength of the correlation suggests that structural improvement explains only a limited proportion of visual acuity changes. In practical terms, this means that even though the retina gets thinner, this does not automatically imply better vision. The reason why retinal anatomical changes were not fully translated to functional gains could be due to damage of photoreceptor integrity, chronicity of the disease, retinal pigment epithelium (RPE) damage and/or fibrosis. This discrepancy between retinal thickness and VA improvement has been previously demonstrated in multiple reports [39,40,41,42].

Fasler and coworkers, in a single-center retrospective study including around 3400 eyes, proved a clear relationship between visual gains and the age determinant with satisfactory resolution [43]. More specifically, they found that younger patients benefit significantly more in terms of visual gains compared to older patients during anti-VEGF therapy and the decreasing trend in the visual gains is proportional to the increase in age. This is also reflected in our data (Figure 4A), where younger age groups appeared to demonstrate slightly greater reductions in logMAR values over time with brolucizumab injections, whereas the oldest subgroup showed comparatively smaller changes. However, these observations should be interpreted cautiously given the smaller number of patients in some age categories and the descriptive nature of this subgroup analysis. Due to the limited sample size within some age strata, no formal between-group statistical comparisons were performed. Overall, based on the final results from this study, low BCVA at presentation, treatment-naïve eyes, and age appear to be the main determinants affecting visual gains after brolucizumab therapy.

Brolucizumab has demonstrated good efficacy in the treatment of nAMD; however, its safety profile has been a matter of debate due to reports of serious inflammatory adverse events. In the pivotal HAWK and HARRIER trials, the overall incidence of adverse events was comparable to aflibercept, but a higher rate of IOI was observed in the brolucizumab group [12]. Later studies identified a range of inflammatory complications including IOI, retinal vasculitis, and retinal vascular occlusion, some of which were associated with clinically significant vision loss [44]. Real-world data have confirmed the occurrence of these events in routine clinical practice, although their overall incidence appears relatively low and most cases occur within the early treatment phase [45,46]. In addition to these drug-specific inflammatory complications, brolucizumab shares the general adverse effects of anti-VEGF therapy, including endophthalmitis, transient intraocular pressure elevation, and conjunctival hemorrhage. In our study, no cases of IOI and/or retinal vasculitis or occlusion were encountered. Most likely, this was due to the stringent inclusion criteria we applied, such that patients with other ophthalmic diseases, patients with previous history of retinal surgery, and other systemic vasculitis were excluded from the study cohort. Nevertheless, we acknowledge that the favorable safety profile observed in our cohort may be influenced by the relatively small sample size and the short duration of follow-up. Similar real-world studies with clean safety profiles involving brolucizumab regarding serious adverse events are reported in the literature [47]. Hence, it turns out that careful patient selection is quite an important paradigm to ensure safe brolucizumab therapy. However, cases of general adverse effects such as sporadic conjunctival bleeding and iritis as well as consistent IOP increase (Figure 4) after each IVI are documented in this study.

The main limitation of this study is the relatively short follow-up duration of 6 months. Although early anatomical and functional responses to anti-VEGF therapy can be observed within the first months of treatment, long-term visual outcomes often require extended monitoring, as visual acuity may stabilize or decline over time depending on treatment adherence and disease progression. Therefore, longer-term studies with follow-up of 2 years or more are needed to better evaluate the sustainability of visual outcomes. Another limitation of this study is the absence of more detailed baseline characterization, such as lesion subtype and duration of symptoms, as well as a lack of the systematic records of fluid characteristics (subretinal and/or intraretinal fluid rates), which may influence treatment response and dosing intervals and thus limit the generalizability of the findings. In addition, lifestyle and systemic factors such as diabetes status and alcohol consumption were not systematically recorded, which may limit the relevance of the findings to a broader scientific context. Moreover, the absence of a control or comparator group precludes direct comparison with other anti-VEGF therapies, while inference of real-world data from a single-center study may be subject to selection bias and may not fully represent broader patient populations. Nevertheless, the study provides valuable information on the early real-world response to brolucizumab therapy in treatment-naïve eyes with low baseline visual characteristics.

## 5. Conclusions

In conclusion, the present study demonstrates that brolucizumab therapy provides significant short-term anatomical and functional benefits in treatment-naïve patients with nAMD and poor baseline visual acuity. Pronounced reductions in central subfield thickness together with meaningful improvements in BCVA were observed, with most visual gains occurring during the loading phase of treatment. Although significant gains in BCVA were achieved, final visual acuity remained modest, likely due to poor baseline vision. This highlights the critical importance of early diagnosis and timely initiation of anti-VEGF therapy. Baseline visual acuity, treatment-naïve status, and patient age appeared to be major determinants affecting visual improvement in this cohort. Although these findings support the early effectiveness of brolucizumab in real-world clinical practice, longer follow-up studies as well as head-to-head studies with other agents such as faricimab are required to define long-term visual prognosis and facilitate anti-VEGF treatment procedures.

## Figures and Tables

**Table 1:** Demographic and baseline characteristics of the study population.

Variable	Value
Age, years (mean ± SD)	68.67 ± 8.29
Sex, *n* (%)	Male: 93 (62.0%) Female: 57 (38.0%)
Eye involved, *n* (%)	Right: 73 (48.7%) Both: 4 (2.6%)
Smoking status, *n* (%)	Yes: 56 (37.3%) No: 94 (62.7%)
Hypertension, *n* (%) ^1^	89 (59.3%)
Hypercholesterolemia, *n* (%) ^1^	95 (63.3%)
Baseline BCVA, logMAR (mean ± SD)	1.26 ± 0.36 (95% CI: 1.20–1.31)
Baseline CST, µm (mean ± SD)	476.6 ± 117.7 (95% CI: 457.6–495.6)

^1^—Hypertension and hypercholesterolemia were defined based on documented diagnosis in medical records and/or current use of corresponding medications.

**Table 2:** Functional (BCVA, logMAR) and anatomical (CST) changes after brolucizumab treatment.

Comparison	ΔBCVA (95% CI)	*p* (VA, Holm)	ΔCST (µm) (95% CI)	*p* (CST, Holm)
Baseline-W4	0.410 (0.372–0.449)	<0.0001	45.5 (39.4–51.6)	<0.0001
Baseline-W8	0.655 (0.607–0.702)	<0.0001	78.5 (69.5–87.5)	<0.0001
Baseline-W12	0.805 (0.754–0.856)	<0.0001	117.8 (105.3–130.3)	<0.0001
Baseline-M6	0.745 (0.699–0.791)	<0.0001	143.6 (128.6–158.6)	<0.0001
W4-W8	0.244 (0.221–0.267)	<0.0001	32.9 (28.7–37.1)	<0.0001
W4-W12	0.395 (0.365–0.424)	<0.0001	72.3 (64.1–80.5)	<0.0001
W4-M6	0.335 (0.290–0.379)	<0.0001	98.1 (87.3–108.9)	<0.0001
W8-W12	0.150 (0.135–0.166)	<0.0001	39.4 (34.0–44.8)	<0.0001
W8-M6	0.090 (0.049–0.132)	<0.0001	65.1 (57.4–72.8)	<0.0001
W12-M6	−0.060 (−0.101–(−0.019))	0.0042	25.7 (20.9–30.5)	<0.0001

Values represent estimated marginal mean differences from repeated-measures ANOVA. Positive ΔBCVA values indicate improvement in visual acuity. Positive ΔCST values indicate reduction in central subfield thickness. Confidence intervals are 95%. *p* values were adjusted using the Holm–Bonferroni method. Abbreviations: W (week), M (month).

## Data Availability

The data used and analyzed for this study are available from the corresponding author on reasonable request.
